# Longitudinal Analysis of Male Fertility Using an Acr‐Luc Knock‐In Mouse Model: A Preclinical Platform for Reproductive Toxicity Testing

**DOI:** 10.1002/mco2.70568

**Published:** 2026-01-04

**Authors:** Hisanori Fukunaga, Ryosuke Seino, Yusuke Matsuya, Hiroyuki Takashima, Masayori Ishikawa, Yasuhito Onodera, Hiroki Shirato, Haruhiko Miyata, Kevin M. Prise

**Affiliations:** ^1^ Department of Biomedical Science and Engineering Faculty of Health Sciences Hokkaido University Sapporo Hokkaido Japan; ^2^ Center For Environmental and Health Sciences Hokkaido University Sapporo Hokkaido Japan; ^3^ Nuclear Science and Engineering Center Japan Atomic Energy Agency Tokai Ibaraki Japan; ^4^ Global Center for Biomedical Science and Engineering Faculty of Medicine Hokkaido University Sapporo Hokkaido Japan; ^5^ Research Institute for Microbial Diseases The University of Osaka Suita Osaka Japan; ^6^ Johnston Cancer Research Centre Queen's University Belfast Belfast UK

**Keywords:** Acr‐Luc knock‐in mouse, bioluminescence imaging, male fertility, reproductive toxicity, spermatogenesis

## Abstract

Reproductive toxicity testing is essential for evaluating whether xenobiotics, including pharmaceuticals, environmental chemicals, or ionizing radiation, adversely affect reproductive function. However, conventional assessments rely on mating outcomes or histopathology, which are labor‐intensive, variable, and require large numbers of animals. Acrosin, a serine protease encoded by the *Acr* gene and localized in the acrosome of spermatozoa, plays a critical role in sperm penetration of the zona pellucida. To exploit this germ cell‐specific expression, we generated a genetically engineered mouse model in which the *Luciferase* (*Luc*) reporter gene is driven by the Acr promoter. This Acr‐Luc knock‐in (KI) model enables longitudinal and quantitative imaging of spermatogenesis using bioluminescence. We demonstrate that this platform captures radiation‐induced impairments in male fertility in real time, eliminating the need for terminal analyses. By allowing repeated evaluation within the same individuals, our approach reduces interindividual variability and enables a substantial reduction in animal use, aligning with the “Reduction” principle of the 3Rs. Moreover, it reveals both the onset and recovery phases of spermatogenic disruption with high temporal resolution. The Acr‐Luc KI model provides a reliable preclinical platform for reproductive toxicity testing and offers broad utility for studies in reproductive biology, toxicology, and oncofertility research.

## Graphical abstract

This study presents an Acr‐Luc knock‐in mouse model that enables longitudinal visualization of spermatogenesis using bioluminescence imaging. By combining a germ cell‐specific promoter with luciferase reporting, the system captures real‐time suppression and recovery of male reproductive function after toxic exposures. The model offers a rapid, sensitive, and animal‐efficient platform for reproductive toxicity assessment and mechanistic studies.

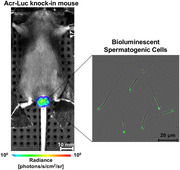

## 1 Introduction

Spermatogenesis is a tightly regulated biological process essential for male fertility and genetic transmission to the next generation. Thus, evaluating whether xenobiotics such as pharmaceuticals, food additives, and environmental chemicals, or external stressors such as ionizing radiation, negatively affect spermatogenesis is a crucial aspect of assessing their overall risk to human health. To support this evaluation, the Organization for Economic Co‐operation and Development (OECD) has established a set of internationally accepted test guidelines, including TG 416, 421, and 443. These guidelines provide a standardized framework for testing chemicals and include a wide range of endpoints related to male reproductive health. Similarly, the International Council for Harmonisation of Technical Requirements for Pharmaceuticals for Human Use (ICH), which sets global standards for drug development, has issued the S5(R3) guideline for assessing reproductive and developmental toxicity. This guideline allows for flexible testing strategies tailored to each stage of drug development. It also encourages the use of alternative test methods, such as cell‐based assays for detecting developmental toxicity, when scientifically validated, making the testing process more efficient and ethically responsible [1].

Although the OECD and ICH guidelines differ in their focus—chemical safety versus pharmaceutical safety—they share a common approach centered around mating studies. These studies help ensure that no harmful effects on male reproductive function are missed. Key evaluation points include examining the structure of male reproductive organs (testes and epididymis), measuring sperm quality (such as number and motility), and assessing the success of mating and fertilization [2]. Both frameworks are also aligned with the 3Rs principle in animal research, namely, reduction, replacement, and refinement, aimed at minimizing animal use and suffering [3]. The OECD, for instance, has introduced the extended one‐generation reproductive toxicity study, a streamlined animal testing method designed to evaluate the effects of chemicals on reproduction and development across a single generation, while reducing the number of animals used [4]. Currently, combining animal studies with mechanistic or screening approaches using alternative methods (e.g., in vitro assays and organotypic models) remains a widely accepted strategy; however, these approaches still require large numbers of animals and substantial breeding costs due to the biological variability inherent in mating, fertilization, and birth outcomes [5].

Ionizing radiation has long served as a classical model for studying germ‐cell vulnerability, with early radiobiological work demonstrating a clear hierarchy of radiosensitivity among spermatogenic cells [6]. These foundational observations have shaped modern understanding of testicular radiation responses and continue to inform the International Commission on Radiological Protection (ICRP) discussions on male reproductive protection [7]. Because radiation induces well‐characterized, dose‐dependent depletion of germ cells, it provides a robust benchmark for evaluating new platforms capable of capturing injury and recovery dynamics. For these reasons, X‐ray irradiation was selected in this study as a well‐validated and reproducible method to induce testicular toxicity in a controlled manner.

Acrosin, a serine protease encoded by the *Acr* gene [8, 9, 10], plays an essential role in sperm–oocyte interaction and successful fertilization. This enzyme is localized within the acrosome, a specialized organelle in the head of spermatozoa, and facilitates sperm penetration through the zona pellucida, the outer glycoprotein layer surrounding the egg [11, 12]. The *Acr* gene is expressed specifically in the testis, and its expression is tightly regulated during spermatogenesis [13, 14]. In mice, *Acr* mRNA has been detected at the premeiotic diploid stage, indicating its involvement from early phases of germ cell maturation [15]. Using a transgenic mouse model expressing green fluorescent protein (GFP) under the control of the Acr promoter [16], we previously examined the impact of radiation on spermatogenesis [17, 18, 19, 20]. While this Acr‐GFP transgenic mouse model has proven valuable for studying cellular responses to environmental challenges, it has a critical limitation: GFP fluorescence does not penetrate biological tissues effectively, making it unsuitable for in vivo imaging applications.

To address the limitations of existing models, we established a novel mouse model that enables longitudinal assessment of the loss and recovery of male reproductive function in individual subjects through BLI. In this study, we generated a knock‐in (KI) mouse model by inserting a transgene construct, comprising the Acr promoter and the *Luciferase* (*Luc*) gene, into the ROSA26 locus [21], a genomic site known for its ubiquitous transcriptional activity (Figure S1). Notably, mice homozygous for ROSA26 modifications are generally healthy and do not exhibit any overt phenotypic abnormalities [22]. This Acr‐Luc KI mouse model was designed to overcome key limitations of conventional reproductive toxicity testing, which relies on mating outcomes or histopathology that are labor intensive, variable, and restricted to terminal analyses. By driving luciferase expression with the germ cell‐specific Acr promoter, the model allows noninvasive monitoring of spermatogenic activity and its disruption by toxic exposures. To demonstrate the utility of this approach, reproductive toxicity was induced using X‐ray irradiation as a controlled and well‐characterized test condition. Our results show that the Acr‐Luc KI mouse model captures both the suppression and recovery phases of spermatogenesis following irradiation, providing a reliable preclinical platform for reproductive biology, toxicology, and oncofertility research.

## 2 Results

### 2.1 Establishment and Characterization of the Acr‐Luc KI Mouse Model

**FIGURE 1:**
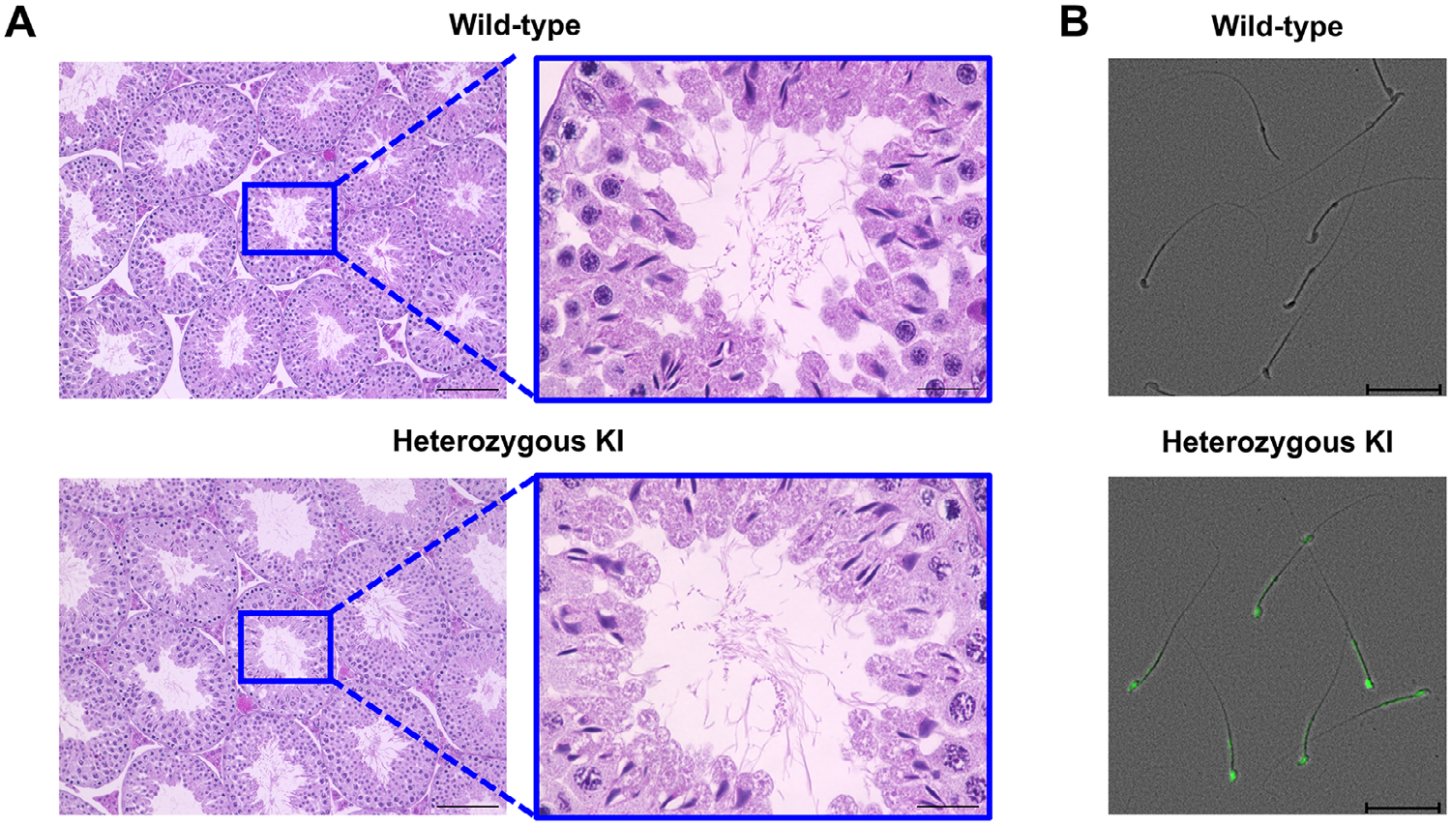
Histological analysis of testis and luciferase imaging of spermatozoa in Acr‐Luc KI mice. (A) Representative images of PAS‐stained testicular sections from Acr‐Luc KI mice. Left: 20× magnification (scale bar: 100 µm); Right: 100× magnification (scale bar: 20 µm). (B) Optical imaging of spermatozoa from wild‐type and Acr‐Luc KI mice following d‐luciferin treatment. Spermatozoa were fragmented in phosphate‐buffered saline (PBS) and treated with d‐luciferin (final concentration: 100 µg/mL). Scale bar: 20 µm (black).

Genotyping by PCR and Southern blotting confirmed the successful integration of the Acr‐Luc construct into the ROSA26 locus (Figures S2–S4). Both heterozygous and homozygous Acr‐Luc KI mice were viable and fertile, with no overt developmental abnormalities. Hematological and biochemical parameters showed no significant differences compared with wild‐type controls (Tables 1 and 2). Magnetic resonance imaging (MRI) analysis confirmed no apparent morphological abnormalities in the testes or epididymis (Figure S5). As shown in Figure 1, histological examination by periodic acid–Schiff (PAS) staining demonstrated normal spermatogenesis in Acr‐Luc KI testes, and bioluminescent signal was detected in mature spermatozoa. Epididymal spermatozoa from Acr‐Luc KI mice displayed normal motility and fertilization capacity, further confirming that introduction of the Acr‐Luc reporter did not impair male reproductive function.

**TABLE 1:** Hematological analysis of Acr‐Luc KI mice.

Test parameters		Wild‐type		Acr‐Luc KI		*t*‐Test
		Average	SD	Average	SD	
WBC	(/µL)	5667	2811	6167	2743	0.836
Neutrophils	(/µL)	663.3	383.7	470.0	124.9	0.453
Lymphocytes	(/µL)	4640	2190	5363	2378	0.718
Monocytes	(/µL)	113.3	37.86	56.67	25.17	0.097
Eosinophils	(/µL)	260.0	238.1	293.3	255.4	0.877
Basophils	(/µL)	10.00	0.000	13.33	5.774	0.374
Neutrophils	(%)	11.57	2.483	8.067	1.966	0.128
Lymphocytes	(%)	82.03	3.099	86.50	2.862	0.141
Monocytes	(%)	2.200	1.127	0.933	0.321	0.135
Eosinophils	(%)	4.000	1.852	4.333	2.274	0.854
Basophils	(%)	0.133	0.058	0.167	0.058	0.519
RBC	(×10^4^/µL)	948.0	73.65	967.00	63.85	0.753
Hemoglobin	(g/dL)	14.73	1.201	15.07	0.764	0.706
Hematocrit	(%)	56.17	1.955	58.43	1.940	0.227
MCV	(fL)	59.43	3.745	60.50	2.524	0.703
MCH	(pg)	15.57	0.153	15.60	0.200	0.830
MCHC	(%)	26.23	1.893	25.80	0.819	0.734
Reticulocyte count	(×10^3^/µL)	321.0	29.11	299.7	59.42	0.607
PLT	(×10^4^/µL)	79.20	44.22	111.30	6.798	0.282

**TABLE 2:** Biochemical analysis of Acr‐Luc KI mice.

Test parameters		Wild‐type		Acr‐Luc KI		*t*‐Test
		Average	SD	Average	SD	
Total protein	(g/dL)	4.867	0.231	5.367	0.321	0.094
Albumin	(g/dL)	3.233	0.208	3.400	0.173	0.346
Total bilirubin	(mg/dL)	>0.1		>0.1		
AST (GOT)	(U/L)	127.0	36.76	132.7	63.81	0.900
ALT (GPT)	(U/L)	56.67	24.58	37.00	13.11	0.289
LDH	(U/L)	784.3	174.8	771.7	164.1	0.931
ALP	(U/L)	125.3	4.509	124.0	4.359	0.731
Amylase	(U/L)	2748	161.1	2672	206.2	0.643
Lipase	(U/L)	49.67	4.726	82.00	63.27	0.427
BUN	(mg/dL)	25.77	0.907	24.23	1.739	0.247
Creatinine	(mg/dL)	0.123	0.006	0.117	0.006	0.230
Triglycerides	(mg/dL)	62.33	9.074	51.33	9.292	0.216
Sodium (Na)	(mEq/L)	151.0	1.732	154.0	2.646	0.176
Potassium (K)	(mEq/L)	9.200	0.436	8.567	0.611	0.218
Chloride (Cl)	(mEq/L)	110.0	1.732	108.0	1.000	0.158
Calcium (Ca)	(mg/dL)	9.500	0.361	9.933	0.569	0.327
Inorganic phosphate	(mg/dL)	10.93	0.153	10.77	0.379	0.519
Blood glucose	(mg/dL)	164.7	34.79	158.0	7.211	0.761
Total bile acids	(µmol/L)	7.067	5.250	30.80	46.70	0.431

### 2.2 Bioluminescence Imaging of Spermatogenesis in Acr‐Luc KI Mice

**FIGURE 2:**
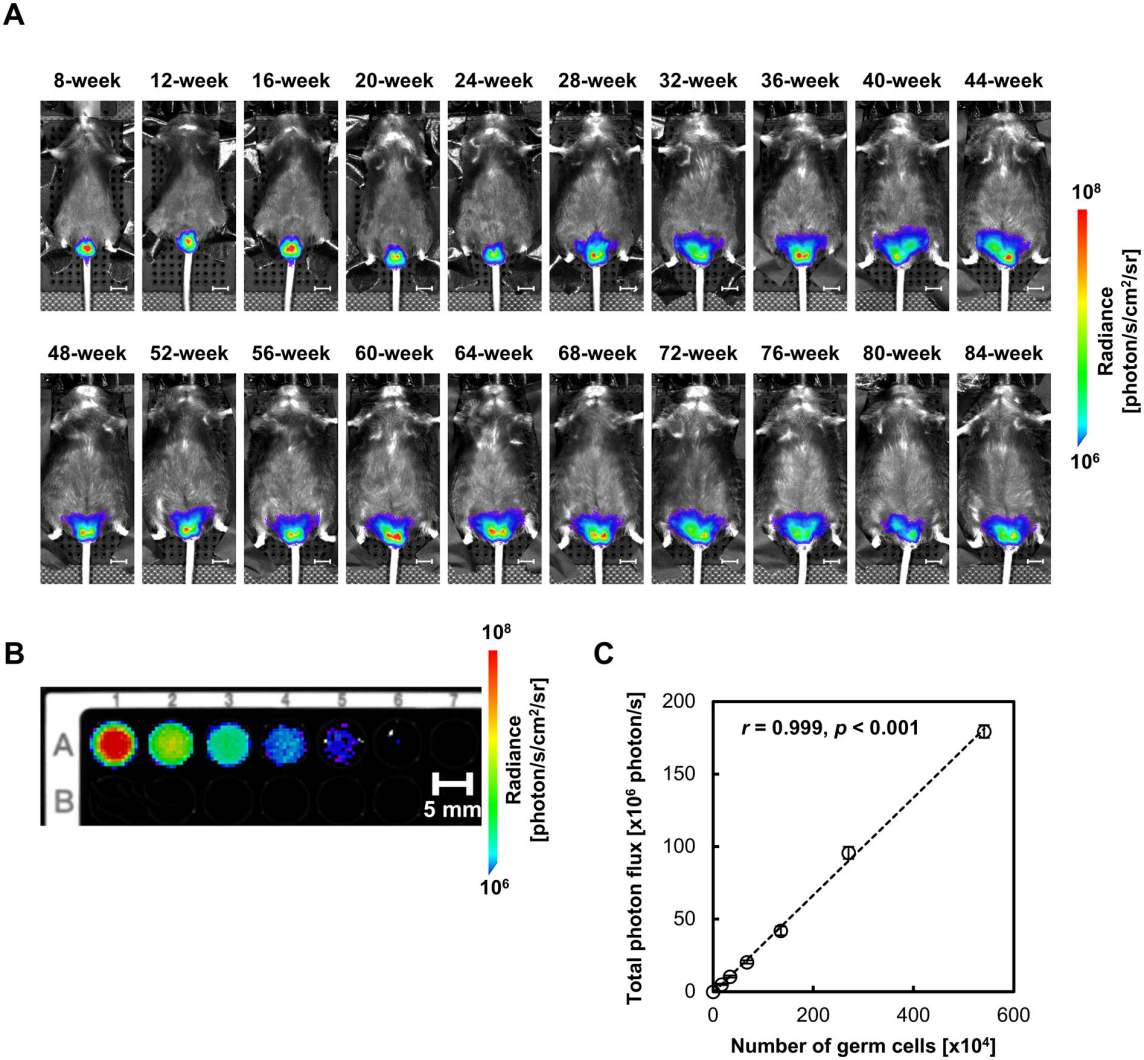
Bioluminescence imaging of spermatogenesis in Acr‐Luc KI mice. (A) Representative time‐course bioluminescence images of a male Acr‐Luc KI mouse. Images were acquired every 4 weeks, starting from 8 weeks of age. Scale bar: 1 cm. (B) In vitro bioluminescence imaging of spermatogenic cells isolated from an Acr‐Luc KI mouse. The cell suspension was serially diluted and plated in a 96‐well black microplate. The relative concentrations from left to right are 1, 1/2, 1/4, 1/8, 1/16, 1/32, and 0 (control). Scale bar: 5 mm. (C) Correlation between the number of germ cells and the corresponding luminescence intensity. The dashed line represents the linear regression line. Pearson's correlation coefficients (*r*) were calculated, with significance set at *p* < 0.05.

**FIGURE 3:**
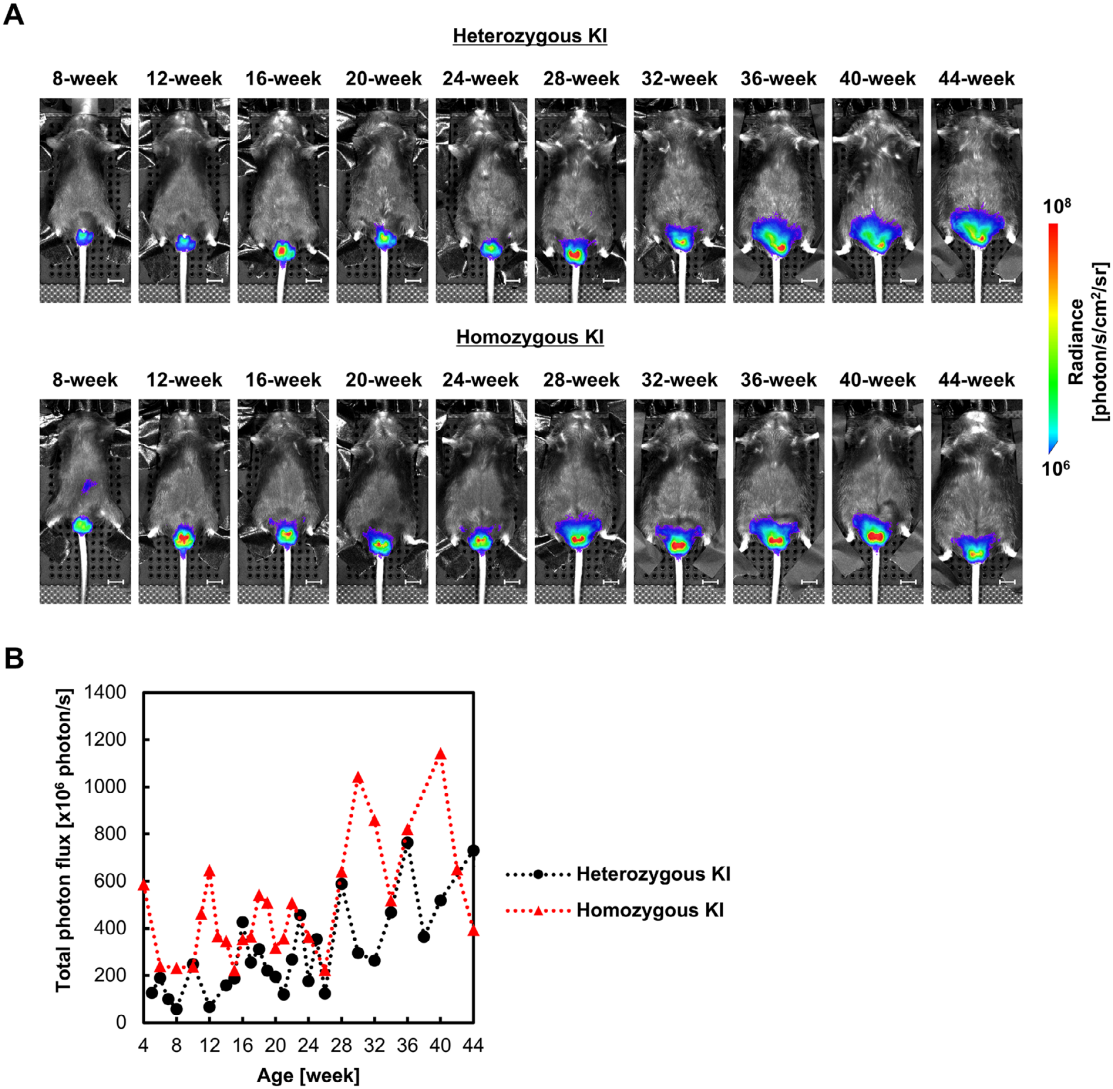
Imaging analysis of heterozygous and homozygous Acr‐Luc KI mice. (A) Representative 2D bioluminescence images of heterozygous and homozygous Acr‐Luc KI mice. Scale bar: 10 mm. (B) Longitudinal analysis of testicular bioluminescence intensity in heterozygous and homozygous Acr‐Luc KI mice at 4‐week intervals from 8 to 44 weeks of age.

Longitudinal BLI demonstrated age‐dependent changes in testicular bioluminescence intensity in Acr‐Luc KI mice (Figure 2A). As shown in Figure 2B,C, the number of spermatogenic cells (from spermatocytes onward) collected by a standardized mechanical dissociation method [23] (Figure S6) was positively correlated with bioluminescence intensity (*r* = 0.999, *p* < 0.001). These indicate that the signal trajectory reflects the progression of spermatogenesis, consistent with histological findings at sexual maturity. In addition, 3D BLI confirmed that the detected luminescence was localized to the testes (Figure S7), verifying that the signal originated from spermatogenic cells. As shown in Figure 3, both heterozygous and homozygous Acr‐Luc KI mice maintained stable luminescence intensity up to 44 weeks of age, providing a sufficient observation window for reproductive toxicity testing. Across longitudinal measurements, luminescence signals exhibited low intra‐animal variability, indicating that Acr‐driven BLI provides a stable and reproducible readout of spermatogenic activity. Importantly, no spontaneous fluctuations exceeding normal experimental variation were detected, supporting the suitability of this system for quantitative temporal tracking of testicular function. Taken together, these results demonstrate that the Acr‐Luc KI model enables longitudinal and quantitative visualization of spermatogenesis in vivo.

### 2.3 Radiation‐Induced Reproductive Toxicity and Recovery Dynamics

**FIGURE 4:**
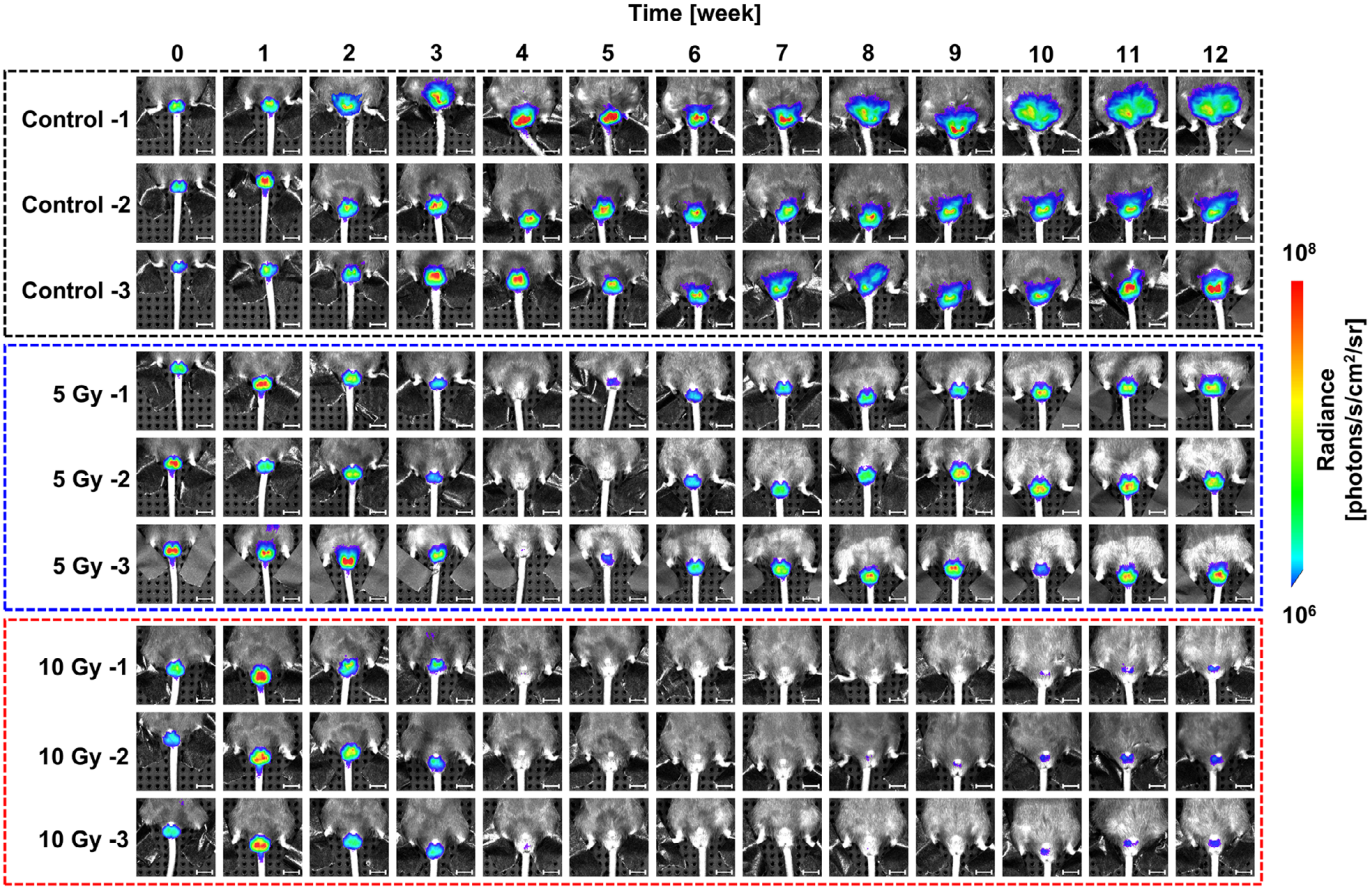
Longitudinal analysis of bioluminescence in control, 5 Gy‐irradiated, and 10 Gy‐irradiated Acr‐Luc KI mice. Representative images of the control, 5 Gy‐irradiated, and 10 Gy‐irradiated Acr‐Luc KI mice. Scale bar: 10 mm.

To evaluate the utility of the Acr‐Lu*c* KI model in reproductive toxicity testing, we exposed the lower body of male mice (4–5 weeks of age) to localized X‐ray irradiation (0, 5, or 10 Gy). Longitudinal BLI revealed a dose‐dependent suppression of spermatogenesis, with complete signal loss observed in both irradiated groups by 4 weeks postirradiation (Figure 4). In control mice, luminescence intensity gradually increased over time, reflecting the normal age‐dependent progression of spermatogenesis. In the 5 Gy group, luminescent signals gradually recovered, reaching levels comparable to controls by 8–12 weeks postirradiation. This recovery was consistent with restored fertility, as confirmed by successful mating with nonirradiated females (Figure S8). In contrast, the 10 Gy group exhibited no recovery of luminescence throughout the observation period, consistent with previous reports demonstrating that 10 Gy X‐irradiated mice become infertile for prolonged periods, with recovery of spermatogenesis requiring activation of dormant Setd4+ spermatogonial stem cells [24].

**FIGURE 5:**
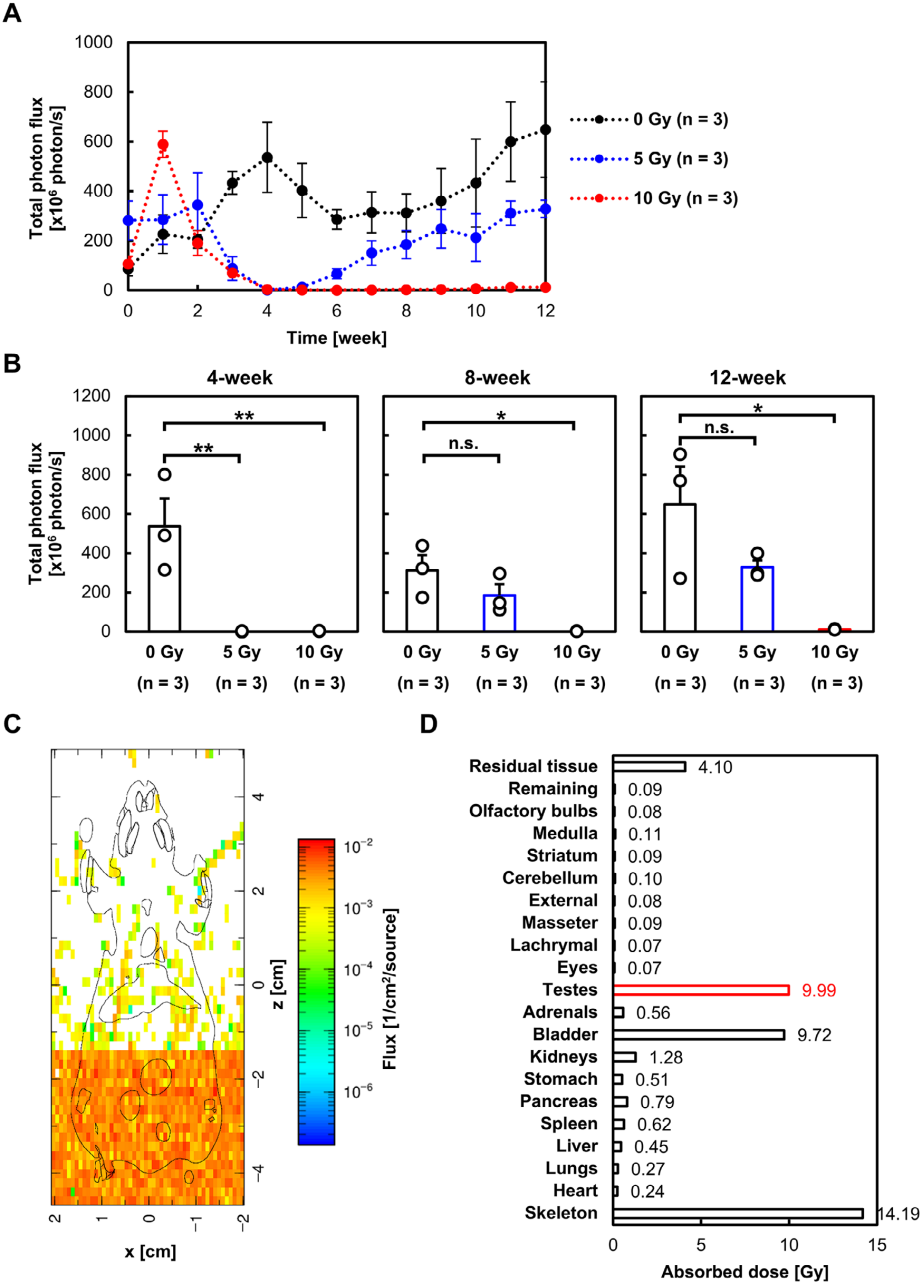
Reproductive toxicity induced by X‐ray irradiation and subsequent recovery. (A) Temporal changes in bioluminescence intensity following localized X‐ray exposure to the lower body. Data are presented as mean total photon flux ± SE. (B) Comparison of bioluminescence intensity among mice irradiated with 0, 5, and 10 Gy, along with representative 2D bioluminescence images. Data are shown as mean total photon flux ± SE with individual data points indicated by white dots. Scale bar: 10 mm. *p < 0.05, **p < 0.01. (C) Dose distribution calculated by PHITS. The 2D distribution of dose is shown for a 15‐week‐old mouse phantom (mesh‐type mouse phantom named “Digimouse”) exposed to 10 Gy at 150 kVp X‐rays. (D) Organ‐specific absorbed dose calculated by PHITS. The absorbed dose in the testes was 10 Gy upon 10 Gy irradiation.

As shown in Figure 5A,B, bioluminescent signals were completely lost by 4 weeks postirradiation, indicating depletion of *Acr*‐expressing spermatogenic cells. By 8 and 12 weeks postirradiation, no significant difference in luminescence intensity was observed between the 0 and 5 Gy groups (*p* = 0.301 and *p* = 0.192, respectively), whereas the 10 Gy group remained suppressed.

To further characterize the irradiation conditions, we quantified organ doses using the particle and heavy ion transport code system (PHITS; version 3.34) [25] coupled with the mesh‐type mouse phantom named “Digimouse” [26, 27]. In the PHITS simulation, the 150 kVp X‐rays were simulated using the electron gamma shower mode [28], and the cutoff energies of both photons and electrons were set to be 1 keV. The energy distribution was sampled using the *t*‐deposit tally, which allows the calculation of deposition energies in certain regions (organs of the Digimouse). The absorbed dose of organs, including testes, was depicted in Figure 5C,D.

## 3 Discussion

Using the Acr‐Luc KI mouse model, we achieved longitudinal and quantitative imaging of spermatogenesis. To our knowledge, this represents the first BLI‐based platform enabling dynamic visualization of male reproductive function in mice. Notably, X‐ray irradiation induced a clear, dose‐dependent suppression and subsequent recovery of male fertility. In contrast to conventional reproductive toxicity assessments, which typically rely on mating studies and terminal histopathological evaluations, both of which require large cohorts of animals and are associated with considerable costs and variability, our imaging‐based model permits repeated, quantitative assessments within the same individuals over time. This approach not only improves temporal resolution in evaluating testicular toxicity and recovery dynamics, but also adheres to the “reduction” principle of the 3Rs by minimizing animal usage and associated husbandry demands. Given these advantages, the Acr‐Luc KI mouse model holds significant promise for broad application in reproductive toxicity testing under OECD and ICH guidelines and may also contribute empirical evidence for future revisions of ICRP recommendations.

A further advantage of our platform derives from the well‐characterized biology of the Acr promoter. Acrosin is expressed exclusively in postmeiotic spermatids, and its transcriptional onset and acrosomal localization have been defined in multiple molecular studies [8, 9, 10, 15]. The essential role of acrosin in zona pellucida penetration has also been demonstrated in both animal and human systems [11, 12]. Because *Acr*‐driven reporters selectively mark this late phase of spermatogenic differentiation, luciferase activity provides a stage‐specific readout of germ‐cell integrity rather than a composite signal integrating multiple developmental steps. The Acr promoter has been widely used in germ‐cell reporter models, including transgenic GFP systems designed to visualize acrosomal maturation [16]. This molecular specificity offers the potential to distinguish toxicants that preferentially target postmeiotic cells from those affecting earlier spermatogenic stages, complementing conventional endpoints that lack cellular resolution. The Acr‐Luc KI mouse may thus serve as a valuable tool for dissecting stage‐dependent vulnerabilities within the spermatogenic lineage.

In addition, the Acr‐Luc KI model may provide valuable insights for the emerging field of oncofertility research. Cancer survivors treated with chemotherapy or radiotherapy often face impaired spermatogenesis and infertility, yet preclinical models to dynamically monitor testicular toxicity and recovery remain limited. Oncofertility is an interdisciplinary field merging oncology and reproductive medicine, aimed at preserving reproductive potential in patients undergoing cancer therapy [29]. By enabling longitudinal imaging of spermatogenic suppression and regeneration, this system could help elucidate the mechanisms of gonadotoxicity, identify protective interventions, and inform fertility preservation strategies in the context of cancer treatment. It could therefore also be applied in research to overcome infertility as an adverse event of chemoradiotherapy, a major challenge in the field of oncofertility [30]. Together, these approaches highlight the value of an in vivo imaging model that can anchor and validate next‐generation in vitro and in silico platforms.

Integration with emerging technologies, particularly artificial intelligence and deep learning‐based image analysis [31], may also enable higher throughput, automated platforms for reproductive toxicity screening. In recent years, the field has been transformed by alternative methodologies such as high throughput in vitro screening platforms (e.g., the Tox21 program) [32], 3D organoid culture systems [33], and CRISPR–Cas9‐based genome editing [34]. These innovations offer human‐relevant mechanistic insights, improve predictive capacity, and reduce animal dependence. Organ‐on‐chip systems and computational modeling approaches further enhance the translational value and efficiency of reproductive toxicity testing [35].

Despite the notable advantages of our model, several limitations should be acknowledged. First, its current applicability is restricted to the evaluation of male reproductive function, precluding its use in the assessment of female reproductive toxicity. Second, the reliable intravenous administration of luciferin requires that experimental animals be at least 3–4 weeks of age, thereby limiting its utility during early developmental stages. This limitation could potentially be addressed through the development of alternative luciferin delivery methods. Third, this approach quantifies spermatogenic activity based on luminescence intensity and therefore may not detect toxicities that do not involve changes in germ cell number. For instance, disruptions in genes critical for fertilization that do not affect cell viability may go undetected using this method, and assisted reproductive technologies may be required to reveal such impairments. Fourth, the current application is confined to mice, and further studies are warranted to adapt and validate this methodology in other rodent species, thereby improving its generalizability. Finally, although we emphasize the preclinical utility of the Acr‐Luc KI mouse model, no mechanistic validation in human tissues or human‐like models has been performed. Therefore, any translational potential should be interpreted with caution, and further investigations using human‐derived cells, organoid systems, or nonrodent models will be required to establish broader applicability.

Beyond radiation biology, the Acr‐Luc KI model also has potential relevance for regulatory reproductive toxicity testing. Conventional frameworks such as the OECD test guidelines (e.g., TG 416, 421, 443) and the ICH S5(R3) guideline for pharmaceuticals rely heavily on mating studies and histopathology, both of which are resource intensive and require large numbers of animals. By enabling repeated, longitudinal assessment of spermatogenesis in the same animals, the Acr‐Luc KI model provides a preclinical platform that could complement and streamline these regulatory approaches. Although further validation will be necessary, particularly across diverse classes of agents, this system could be applied to the evaluation of industrial chemicals, agricultural chemicals, and pharmaceuticals, thereby enhancing both the efficiency and ethical standards of reproductive toxicity testing.

## 4 Conclusion

We established a novel luciferase reporter animal model, the Acr‐Luc KI mouse, which enables longitudinal and quantitative assessment of male fertility with bioluminescence imaging. This platform holds promise not only for mechanistic studies of reproductive toxicity and recovery but also for advancing ethical and efficient toxicological screening by reducing animal use in accordance with the 3Rs.

## 5 Methods

### 5.1 Generation of Acr‐Luc KI Mice

As shown in Figure S2, to generate the novel KI mouse model, a homologous recombination vector was constructed to insert the *Luc* gene under the control of the Acr promoter. The vector was introduced into embryonic stem (ES) cells derived from the C57BL/6N RENKA strain via electroporation. Following drug selection, neomycin‐resistant ES cell colonies were isolated. Genomic DNA was then extracted from individual clones, and homologous recombination events were initially screened by polymerase chain reaction (PCR) (Figure S3). Clones identified as positive by PCR were further validated by Southern blot analysis using a neo probe to confirm correct targeting and to obtain homologous recombinant ES cell lines. Chimeric embryos were generated via the aggregation method, combining homologous recombinant ES cell clones with eight‐cell‐stage embryos derived from the ICR lineage. On the anticipated day of parturition, delivery by recipient female mice was confirmed; in cases where delivery had not occurred spontaneously, cesarean section was performed. The resulting chimeric pups were raised until weaning, and the degree of chimerism was estimated based on coat color.

First‐generation (F1) mice were obtained through natural mating between chimeric mice and wild‐type mice. Genomic DNA extracted from somatic tissues of the offspring was analyzed by PCR to identify germline‐transmitting chimeras. To generate second‐generation (F2) mice, F1 animals were crossed with CAG‐Flp mice (*ROSA26^KI(CAG‐Flp)/KI(CAG‐Flp)^*). Offspring were genotyped by PCR using genomic DNA from somatic tissues, and individuals lacking the *neo* selection cassette (*ROSA26^KI(Acr‐Luc)/KI(CAG‐Flp)^*) were selected. Third‐generation (F3) mice were subsequently produced by natural mating between F2 heterozygous mice (*ROSA26^KI(Acr‐Luc)/KI(CAG‐Flp)^*) and wild‐type mice. Genomic DNA from the F3 progeny was analyzed by PCR to identify and isolate mice lacking the CAG‐Flp allele, resulting in the final target genotype: (*ROSA26^KI(Acr‐Luc)/+^*) (Figure S4). Unless otherwise specified, heterozygous Acr‐Luc KI mice were used for all experiments.

### 5.2 Bioluminescence Imaging Analysis

BLI was performed weekly or biweekly starting at 4–5 weeks of age using the IVIS spectrum computed tomography (CT) system (PerkinElmer, Waltham, MA, USA), equipped with Living Image software. Mice were anesthetized with 1–2% isoflurane (FUJIFILM Wako Pure Chemical Co., Osaka, Japan; #095‐06573) delivered in oxygen via a precision vaporizer, both prior to and during image acquisition. d‐Luciferin potassium salt (OZ Biosciences, Marseille, France; #LK10000) was administered intravenously via the tail vein at a dose of 150 mg/kg. Following injection, mice were placed in a supine position on the imaging stage, and luminescence images were acquired approximately 15 min later. Imaging parameters were standardized across all sessions: 1‐s exposure time, medium binning, and f/1 aperture.

For two‐dimensional (2D) bioluminescence imaging analysis, a 620 nm emission filter was used, and a circular region of interest (ROI) with a diameter of 25 mm was manually placed to encompass the entire light‐emitting area. Additionally, during the first imaging session (at 4 or 5 weeks of age) and again at 12 and 20 weeks of age, optical images were acquired using three emission filters (600, 620, and 640 nm). These images were subsequently integrated with CT scans obtained using the Standard One Mouse protocol to generate three‐dimensional (3D) reconstruction data.

For 3D imaging analysis, a cubic volume of interest (VOI) measuring 25 × 25 × 25 mm was manually positioned to cover the full extent of the bioluminescent signal. For both 2D and 3D analyses, corresponding ROI and VOI were placed in background regions, and background‐subtracted bioluminescent signals were quantified as photons per second. All image acquisition and processing were carried out using Living Image software.

### 5.3 In Vitro Luminescence Imaging

Testicular germ cells were isolated from 4‐week‐old male mice as described [23]. Briefly, testes were excised and the tunica albuginea was removed in an erythrocyte lysing buffer (PluriSelect Life Science, Leipzig, Germany) according to the manufacturer's protocol. Seminiferous tubules were transferred to phosphate‐buffered saline (PBS) containing Ca^2^⁺ and Mg^2^⁺ and supplemented with 5.6 mM glucose, 5.4 mM sodium lactate, 0.1 mg/mL polyvinyl alcohol, and 5 mg/mL bovine serum albumin. The tubules were gently teased apart with fine forceps, minced, and further dissociated by gentle pipetting to release germ cells. The resulting suspension was filtered through a 38 µm nylon mesh. Cells were collected by centrifugation at 400×*g* for 4 min at room temperature, and the pellet was resuspended in PBS. d‐Luciferin (OZ Biosciences) was added to a final concentration of 0.5 mM. The suspension (200 µL per well) was immediately plated into a 96‐well black microplate (Eppendorf, Hamburg, Germany), and luminescence was measured using the IVIS Spectrum CT imaging system.

### 5.4 PAS staining for testicular tissues

Testicular tissues were fixed, embedded in paraffin, and processed for PAS staining. From each paraffin block, tissue sections were prepared, and the section containing the largest cross‐sectional area was selected. This section was stained with PAS and counterstained with Mayer's hematoxylin.

### 5.5 Animal Magnetic Resonance Imaging

MRI in this study was performed using a 7‐Tesla scanner (Pharmascan 70/16; Bruker, Billerica, MA, USA) equipped with a quadrature transceiver volume coil (inner diameter: 72 mm). 3D T1‐weighted images were acquired in the axial plane using a fast low‐angle shot (FLASH) sequence with the following parameters: echo time = 8 ms, repetition time = 50 ms, flip angle = 20°, voxel size = 0.2 × 0.2 × 0.2 mm^3^, and number of signal averages (NSA) = 1. MRI datasets were processed using ZioCube software (version 1.0.2.0; Ziosoft Inc., Tokyo, Japan).

### 5.6 Irradiation Settings

To model temporary and permanent infertility induced by ionizing radiation, male mice were locally irradiated to the lower body, including both hind limbs and the pelvic region, with single doses of 0 (sham‐irradiation), 5, or 10 Gy of 150 kVp X‐rays using an MBR‐1520R‐4 generator (Hitachi Power Solutions, Ibaraki, Japan) equipped with a 1.0‐mm aluminum filter (Figure S9). The dose rate was 1.80 Gy/min, and air kerma was monitored in real time with an ionization chamber. To preserve the integrity of the tail vein used for luciferin administration, the tail was shielded with a lead (Pb) sheet during irradiation.

The Pb shielding profile was validated using Gafchromic RTQA2 film (Ashland Inc., Wayne, NJ, USA; #11032202), as shown in Figure S4B. Irradiated films were scanned 24 h postexposure with a flatbed color scanner (ES‐10000G; Seiko Epson Corp., Nagano, Japan) at 1200 dpi resolution and 32‐bit color depth.

### 5.7 Statistical Analysis

BLI of Acr‐Luc KI mice was performed weekly until 24 weeks of age and biweekly thereafter. For comparison of irradiated groups (5 and 10 Gy) with the 0 Gy group at 4, 8, and 12 weeks postirradiation, Dunnett's test was used. Hematological and biochemical parameters (Tables 1 and 2) were compared between Acr‐Luc KI and wild‐type mice using Student's *t*‐test. The relationship between luminescence intensity and germ cell counts was evaluated using Pearson's correlation coefficient. A two‐sided *p* value of <0.05 was considered statistically significant. All statistical analyses were conducted using Python Anaconda (version 23.7.4).

## Author Contributions

Hisanori Fukunaga: conceptualization, funding acquisition, investigation, methodology, project administration, resources, supervision, writing – original draft preparation, and writing – review and editing. Ryosuke Seino: data curation, investigation, methodology, and software. Yusuke Matsuya: methodology, software, validation, and writing – review and editing. Hiroyuki Takashima: data curation and investigation. Masayori Ishikawa: methodology and validation. Yasuhito Onodera: methodology and validation. Hiroki Shirato: methodology and validation. Haruhiko Miyata: methodology and writing – review and editing. Kevin M. Prise: conceptualization, supervision, and writing – review and editing. All authors have read and approved the final manuscript.

## Funding

This work was supported by JSPS KAKENHI (JP24K03079), JST FOREST Program (JPMJFR211E), and Medical Research Grants from the Takeda Science Foundation. Funding agencies had no role in study design, data collection, analysis, or manuscript preparation.

## Ethics Statement

All animal experiments were conducted in accordance with the Hokkaido University Regulations of Animal Experimentation and were approved by the Institutional Animal Care and Use Committee of the National University Corporation at Hokkaido University (approval number: 23–0031).

## Conflicts of Interest

The authors declare no conflicts of interest.

## Supporting information




**Figure S1**. Schematic representation of the Acr‐Luc ROSA26 targeting vector. This includes a splicing acceptor (SA), the Acr promoter, Luc2, and a bovine growth hormone polyadenylation signal (bGH polyA). Additionally, a neomycin resistance (neo) cassette flanked by FRT sites was incorporated for positive selection. These elements were inserted between the 5' and 3' homology regions.
**Figure S2**. Overview for Acr‐Luc ROSA26 locus knock‐in strategy. (**A**) The 5′ homology arm of the targeting vector included exon 1 of the ROSA26 locus and extended 3.3 kb downstream to the XbaI restriction site. The 3′ homology arm comprised a 4.3 kb region located downstream of the same XbaI site. (**B**) The homologous recombination vector was designed to insert a splicing acceptor (SA), a bovine growth hormone polyadenylation signal (bGH polyA), and a transgene cassette consisting of the Acr promoter, Luc2, and bGH polyA, along with a neomycin resistance (neo) gene flanked by FRT sites, between the 5′ and 3′ homology arms. (**C**) Homologous recombination events were initially screened by PCR using primers located outside the homology arms and within the SA and neo sequences. Positive clones were further validated by Southern blot analysis using a neo‐specific probe. For Southern blotting, genomic DNA was digested with restriction enzymes (NheI and KpnI) that recognize sequences located outside the homology arms to confirm correct integration. (**D**) Final targeted allele after excision of the neomycin resistance cassette.
**Figure S3**. PCR settings for ROSA26 locus knock‐in screening.
**Figure S4**. PCR settings to identify mice lacking the CAG‐Flp allele.
**Figure S5**. Reconstructed MR images centered on the testes of Acr‐Luc KI mouse. (**A**) Transverse plane image. (**B**) Coronal plane image.
**Figure S6**. Brightfield and luminescence imaging of spermatogenic cells. (**A**) A representative brightfield image of isolated spermatogenic cells. (**B**) The corresponding luminescence signal (green, 475–575 nm) from the same field of view. (**C**) Merged image of the brightfield (A) and luminescence (B) signals. Images were acquired using an All‐in‐One BZ‐9000 (Keyence, Osaka, Japan) with a 40× objective lens. The spermatogenic cell suspension was mounted on a glass slide with a coverslip for observation. Yellow, red, and blue arrows indicate a spermatocyte, spermatid, and sperm, respectively. Scale bar: 20 µm.
**Figure S7**. 3D bioluminescence imaging analysis of Acr‐Luc KI mice. (**A**) 3D reconstruction of testicular tissues generated from 2D bioluminescence images. (**B**) Quantification of bioluminescence intensity in VOI at 4, 12, and 20 weeks of age. Data are presented as mean total photon flux ± SE from three mice; white dots indicate individual data points. *n.s*. = *not significant*.
**Figure S8**. Pups of a 5 Gy‐irradiated Acr‐Luc knock‐in mouse. A 5 Gy‐irradiated Acr‐Luc knock‐in mouse (corresponding to the “5 Gy‐3” individual in Figure 4) was mated with a wild‐type female 12 weeks after irradiation. The image shows the three pups born 24 days postmating. No apparent morphological abnormalities or abnormal behaviors were observed in the pups.
**Figure S9**. X‐ray irradiation setup and dose verification for Acr‐Luc KI mice. (**A**) Schematic of the X‐ray irradiation setup targeting the lower body of Acr‐Luc KI mice. To ensure coverage of the testes despite physiological movement, the irradiation field included the region at and below the pelvic bones. Acr‐Luc KI mice were anesthetized with isoflurane and exposed to 150 kVp X‐rays. The exposure to the upper body was shielded using a lead (Pb) block (15.0 × 10.0 × 5.0 cm^3^). (**B**) Radiographic confirmation of the irradiation field using radiochromic film. The outline of the irradiated mouse body is indicated by a red dashed line. Scale bar: 10 mm.

## Data Availability

All data supporting the findings of this study are included in the main text and/or the Supporting Information.
