# Supplementary material for: Longitudinal Analysis of Male Fertility Using an Acr‐Luc Knock‐In Mouse Model: A Preclinical Platform for Reproductive Toxicity Testing
Source: MedComm (2020). 2026 Jan 4;7(1):e70568. doi: 10.1002/mco2.70568 (PMC12765401; doi:10.1002/mco2.70568)
Supplement: Supplementary file 1 — Figure S1. Schematic representation of the Acr‐Luc ROSA26 targeting vector. This includes a splicing acceptor (SA), the Acr promoter, Luc2, and a bovine growth hormone polyadenylation signal (bGH polyA). Additionally, a neomycin resistance (neo) cassette flanked by FRT sites was incorporated for positive selection. These elements were inserted between the 5' and 3' homology regions. Figure S2. Overview for Acr‐Luc ROSA26 locus knock‐in strategy. (A) The 5′ homology arm of the targeting vector included exon 1 of the ROSA26 locus and extended 3.3 kb downstream to the XbaI restriction site. The 3′ homology arm comprised a 4.3 kb region located downstream of the same XbaI site. (B) The homologous recombination vector was designed to insert a splicing acceptor (SA), a bovine growth hormone polyadenylation signal (bGH polyA), and a transgene cassette consisting of the Acr promoter, Luc2, and bGH polyA, along with a neomycin resistance (neo) gene flanked by FRT sites, between the 5′ and 3′ homology arms. (C) Homologous recombination events were initially screened by PCR using primers located outside the homology arms and within the SA and neo sequences. Positive clones were further validated by Southern blot analysis using a neo‐specific probe. For Southern blotting, genomic DNA was digested with restriction enzymes (NheI and KpnI) that recognize sequences located outside the homology arms to confirm correct integration. (D) Final targeted allele after excision of the neomycin resistance cassette. Figure S3. PCR settings for ROSA26 locus knock‐in screening. Figure S4. PCR settings to identify mice lacking the CAG‐Flp allele. Figure S5. Reconstructed MR images centered on the testes of Acr‐Luc KI mouse. (A) Transverse plane image. (B) Coronal plane image. Figure S6. Brightfield and luminescence imaging of spermatogenic cells. (A) A representative brightfield image of isolated spermatogenic cells. (B) The corresponding luminescence signal (green, 475–575 nm) from the same fi [file MCO2-7-e70568-s001.pdf]

Supporting Information for

Longitudinal analysis of male fertility using an Acr-Luc knock-in mouse model: A preclinical platform for reproductive toxicity testing

Hisanori Fukunaga[1,2]*, Ryosuke Seino[1], Yusuke Matsuya[1,3], Hiroyuki Takashima[1], Masayori Ishikawa[1], Yasuhito Onodera[4], Hiroki Shirato[4], Haruhiko Miyata[5], and Kevin M. Prise[6]

[1] Department of Biomedical Science and Engineering, Faculty of Health Sciences, Hokkaido University, N12 W5 Kita-ku, Sapporo, Hokkaido 060-0812, Japan

[2] Center for Environmental and Health Sciences, Hokkaido University, N12 W7 Kita-ku, Sapporo, Hokkaido 060-0812, Japan

[3] Nuclear Science and Engineering Center, Japan Atomic Energy Agency, 2-4 Shirakata, Tokai, Ibaraki 319-1195, Japan

[4] Global Center for Biomedical Science and Engineering, Faculty of Medicine, Hokkaido University, N15 W7 Kita-ku, Sapporo, Hokkaido 060-0815, Japan

[5] Research Institute for Microbial Diseases, The University of Osaka, 3-1 Yamadaoka, Suita, Osaka 565-0871, Japan

[6] Johnston Cancer Research Centre, Queen's University Belfast, 97 Lisburn Road, Belfast BT9 7AE, UK

***Correspondence:** Hisanori Fukunaga, Email: hisanori.fukunaga@hs.hokudai.ac.jp

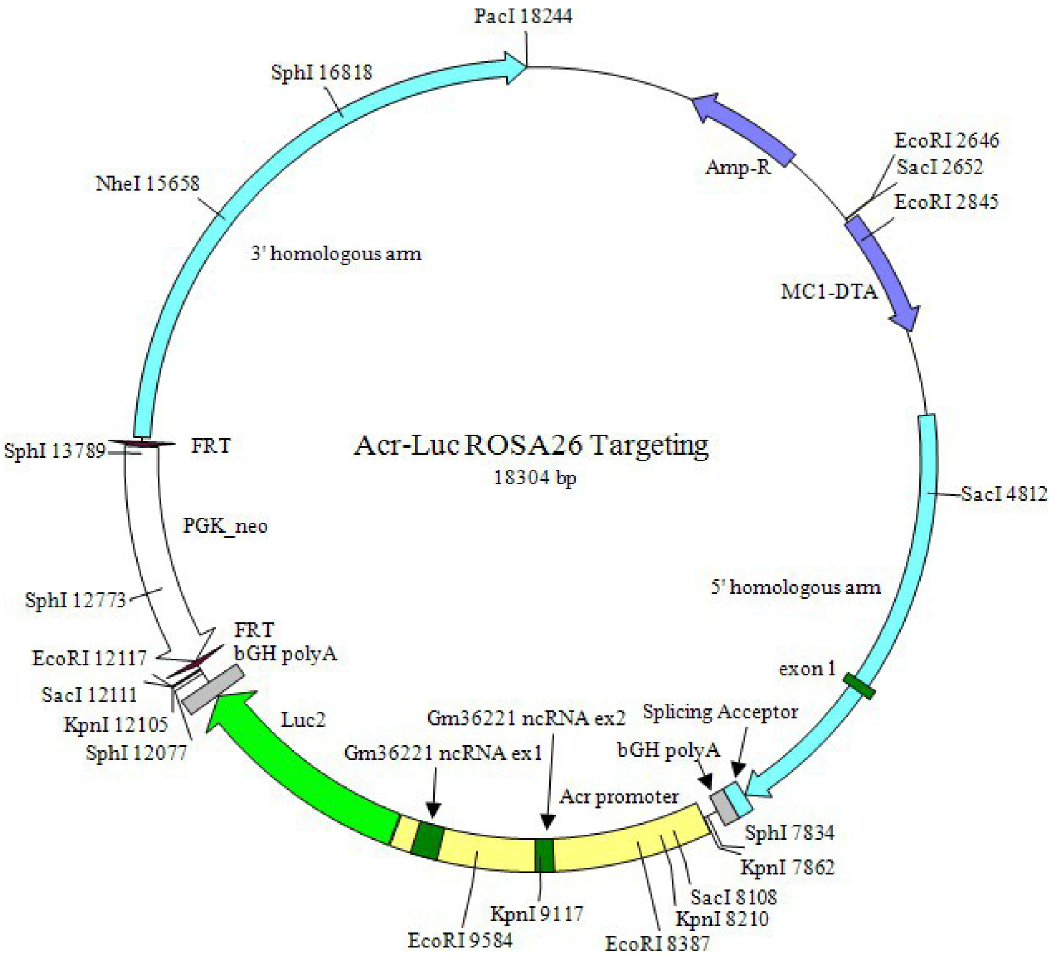

Figure S1. Schematic representation of the Acr-Luc ROSA26 targeting vector.

This includes a splicing acceptor (SA), the Acr promoter, Luc2, and a bovine growth hormone polyadenylation signal (bGH polyA). Additionally, a neomycin resistance (neo) cassette flanked by FRT sites was incorporated for positive selection. These elements were inserted between the 5' and 3' homology regions.

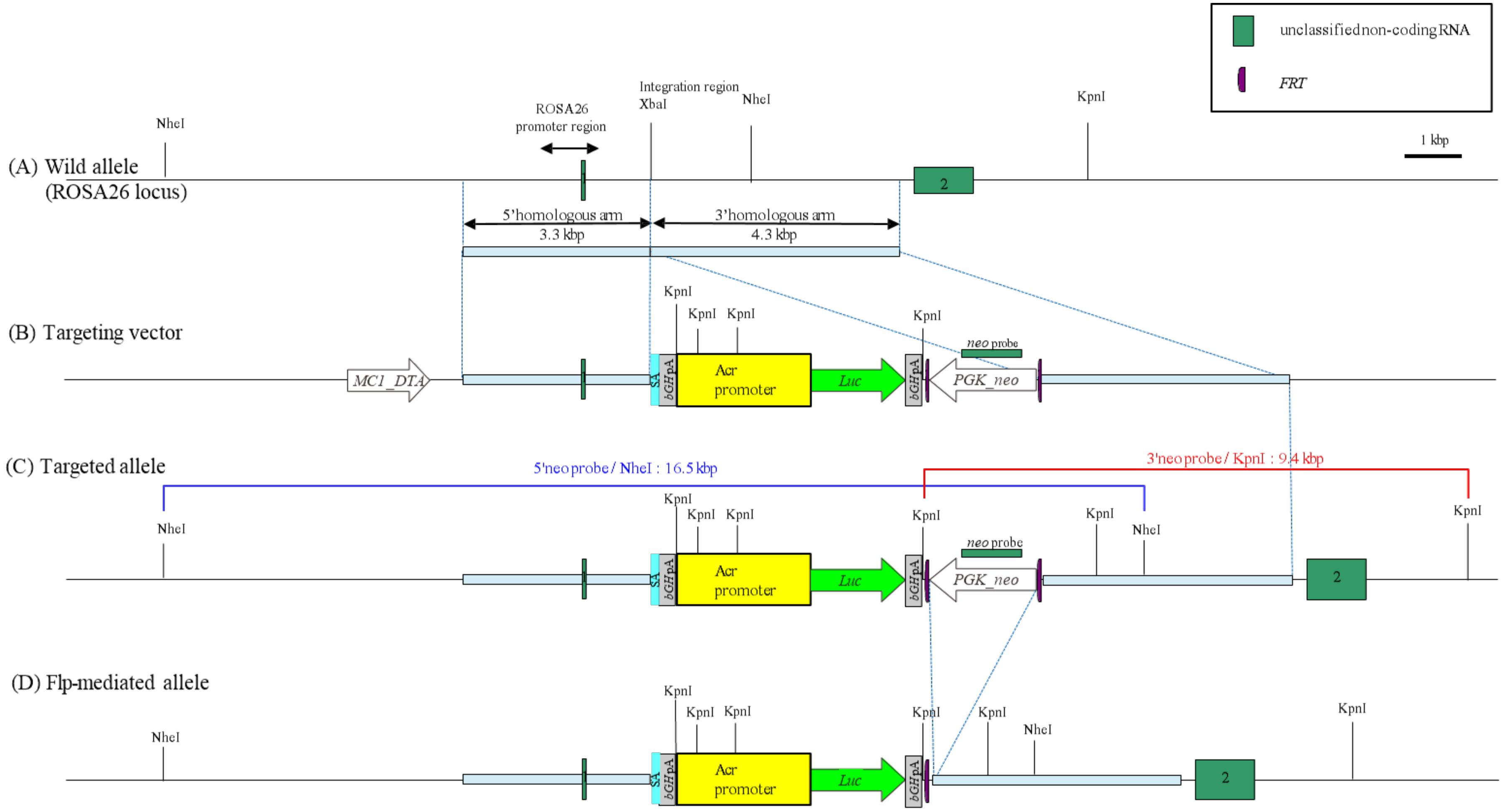

unclassifiednon-codingRNA
FRT
1 kbp
(A) Wild allele
(ROSA26 locus)
NheI
ROSA26
promoter region
Integration region
XbaI
NheI
KpnI
2
5'homologous arm
3.3 kbp
3'homologous arm
4.3 kbp
(B) Targeting vector
MC1_DTA
SA
bGHpA
Acr
promoter
Luc
PGK_neo
neo probe
KpnI
(C) Targeted allele
5'neo probe / NheI : 16.5 kbp
3'neo probe / KpnI : 9.4 kbp
(D) Flp-mediated allele

Figure S2. Overview for Acr-Luc ROSA26 locus knock-in strategy.

(**A**) The 5′ homology arm of the targeting vector included exon 1 of the ROSA26 locus and extended 3.3 kb downstream to the XbaI restriction site. The 3′ homology arm comprised a 4.3 kb region located downstream of the same XbaI site.

(**B**) The homologous recombination vector was designed to insert a splicing acceptor (SA), a bovine growth hormone polyadenylation signal (bGH polyA), and a transgene cassette consisting of the Acr promoter, Luc2, and bGH polyA, along with a neomycin resistance (neo) gene flanked by FRT sites, between the 5′ and 3′ homology arms.

(**C**) Homologous recombination events were initially screened by PCR using primers located outside the homology arms and within the SA and neo sequences. Positive clones were further validated by Southern blot analysis using a neo-specific probe. For Southern blotting, genomic DNA was digested with restriction enzymes (NheI and KpnI) that recognize sequences located outside the homology arms to confirm correct integration.

(**D**) Final targeted allele after excision of the neomycin resistance cassette.

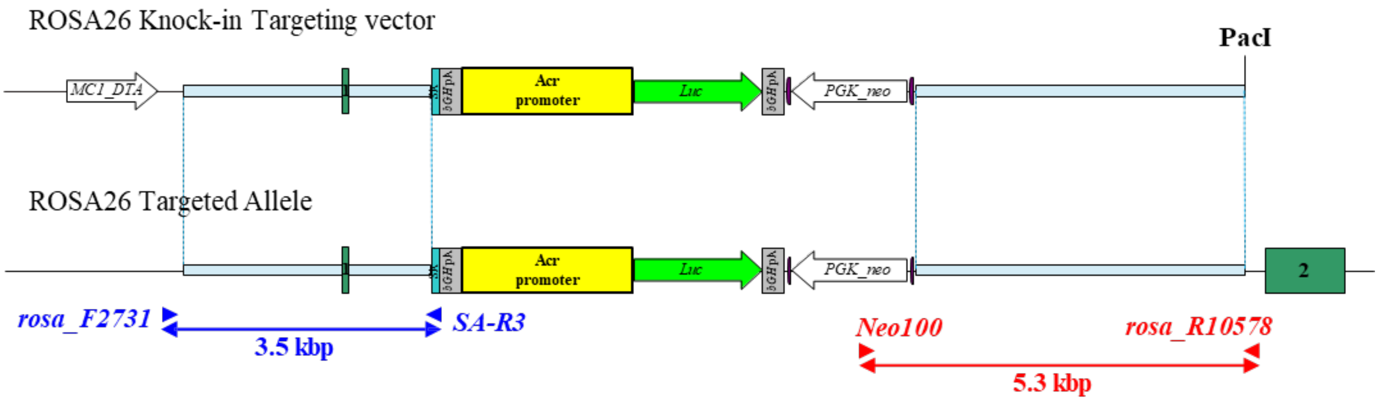

5'PCR Condition

PCR primers	Primer name	Primer sequence				Length
	rosa_F2731	CCATGCTGGAAGGATTGGAACTATGC				26
	SA-R3	AGGATAAGTATGACATCATCAAGG				24
PCR conditions	PCR enzyme	KOD FX (TOYOBO)				
	Thermocycler	GeneAmp PCR System 9700 (Applied Bio Systems)				
	Component	Volume	Cycling condition			3 -step
	template	1.0 µl	Predenature	:	94.0 °C	2 min.
	2×FX buffer	12.5 µl	Denature	:	98.0 °C	10 sec. ※
	2.0 mM dNTPs each	5.0 µl	Annealing	:	62.5 °C	30 sec. ※
	Forward primer (10 µM)	0.75 µl	Extension	:	68.0 °C	4 min. ※
	Reverse primer (10 µM)	0.75 µl	Final extension	:	68.0 °C	4 min.
	KOD FX	0.5 µl	Hold	:	4.0 °C	∞
	ddw	4.5 µl	※Number of cycles			35 cycles
	Total	25.0 µl	Size of PCR products			3.5 kbp

3'PCR Condition

PCR primers	Primer name	Primer sequence				Length
	Neo 100	AGGTGAGATGACAGGAGATC				20
	rosa_R10578	AAGCTTACCATCAACCTTATAGTACAC				27
PCR conditions	PCR enzyme	KOD FX (TOYOBO)				
	Thermocycler	GeneAmp PCR System 9700 (Applied Bio Systems)				
	Component	Volume	Cycling condition			3 -step
	template	1.0 µl	Predenature	:	94.0 °C	2 min.
	2×FX buffer	12.5 µl	Denature	:	98.0 °C	10 sec. ※
	2.0 mM dNTPs each	5.0 µl	Annealing	:	65.0 °C	30 sec. ※
	Forward primer (10 µM)	0.75 µl	Extension	:	68.0 °C	6 min. ※
	Reverse primer (10 µM)	0.75 µl	Final extension	:	68.0 °C	5 min.
	KOD FX	0.5 µl	Hold	:	4.0 °C	∞
	ddw	4.5 µl	※Number of cycles			35 cycles
	Total	25.0 µl	Size of PCR products			5.3 kbp

Figure S3. PCR settings for ROSA26 locus knock-in screening.

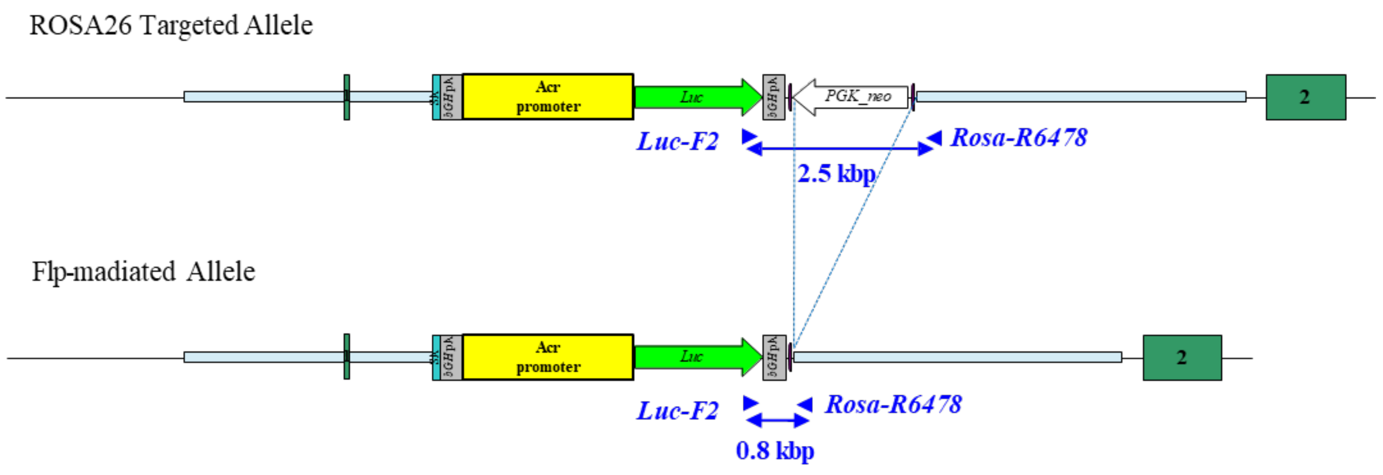

PCR Condition

PCR primers	Primer name	Primer sequence	Length
	Luc-F2	CATGACCGAGAAGGAGATCGTGG	23
	rosa-R6478	AGCATGTCTTTAATCTACCTCGATGG	26

PCR conditions		
	PCR enzyme	KOD FX (TOYOBO)
	Thermocycler	GeneAmp PCR System 9700 (Applied Bio Systems)

Component	Volume
template	1.0 µl
2×FX buffer	12.5 µl
2.0 mM dNTPs each	5.0 µl
Forward primer (10 µM)	0.75 µl
Reverse primer (10 µM)	0.75 µl
KOD FX	0.5 µl
ddw	4.5 µl
Total	25.0 µl

Cycling condition			2 -step	
Predenature	:	94.0 °C	2 min.	
Denature	:	98.0 °C	10 sec.	※
Extension	:	68.0 °C	2.5 min.	※
Final extension	:	68.0 °C	2.5 min.	
Hold	:	4.0 °C	∞	

※Number of cycles	35 cycles
Size of PCR products : Targeted	2.5 kbp
Size of PCR products : Flp-med	0.8 kbp
Size of PCR products : Wild type	-

Figure S4. PCR settings to identify mice lacking the CAG-Flp allele.

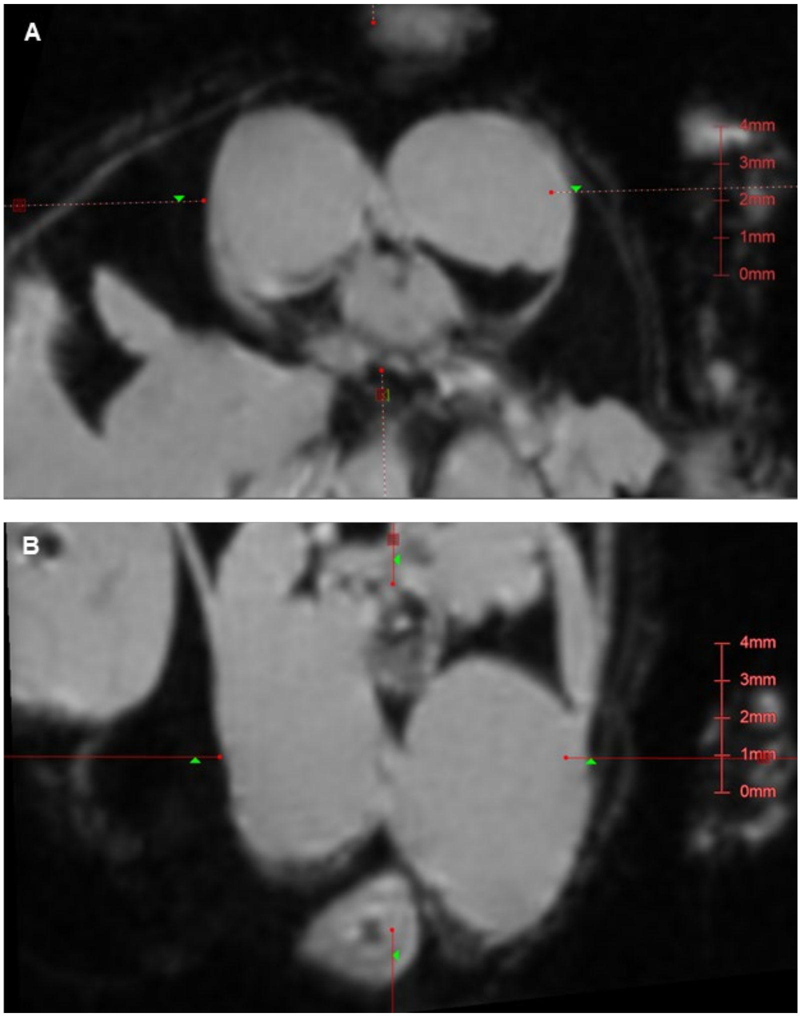

Figure S5. Reconstructed MR images centered on the testes of Acr-Luc KI mouse. (**A**) Transverse plane image. (**B**) Coronal plane image.

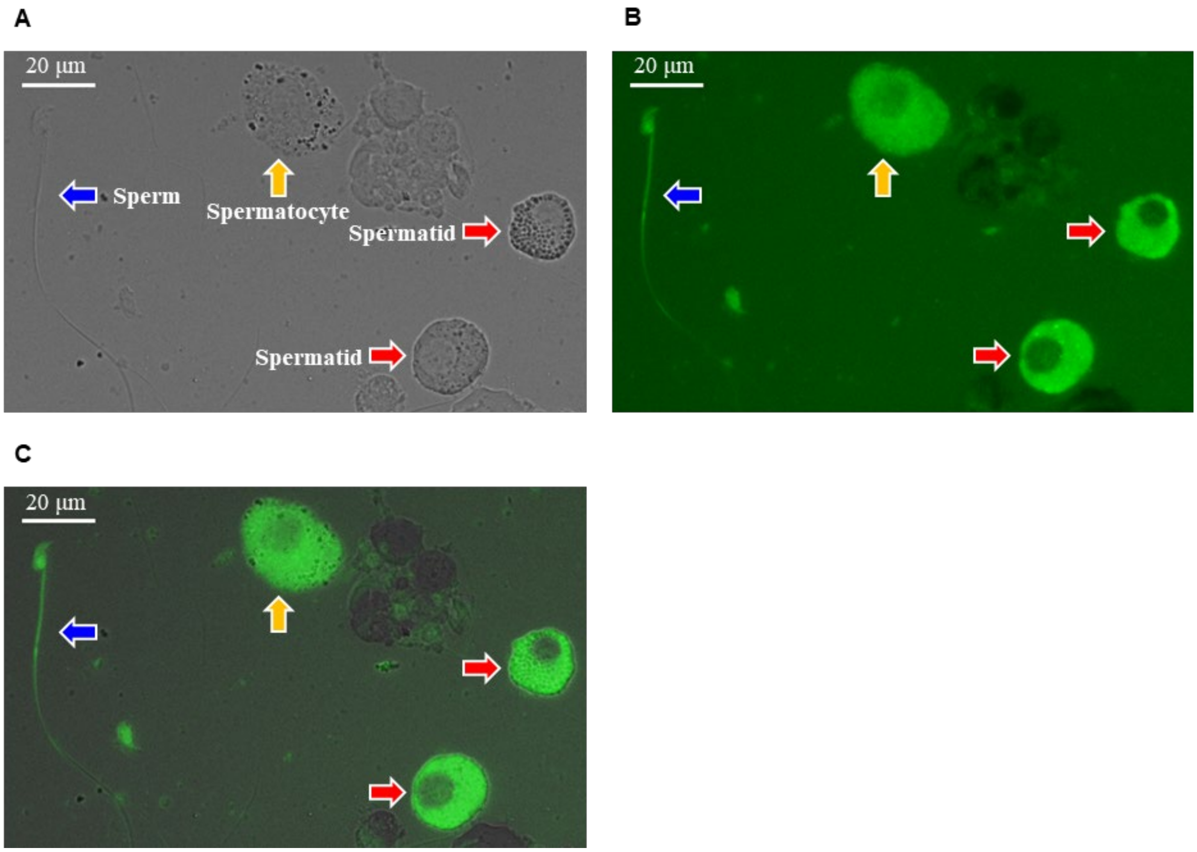

Figure S6. Brightfield and luminescence imaging of spermatogenic cells.

(**A**) A representative brightfield image of isolated spermatogenic cells.

(**B**) The corresponding luminescence signal (green, 475–575 nm) from the same field of view.

(**C**) Merged image of the brightfield (A) and luminescence (B) signals. Images were acquired using an All-in-One BZ-9000 (Keyence, Osaka, Japan) with a 40× objective lens. The spermatogenic cell suspension was mounted on a glass slide with a coverslip for observation. Yellow, red, and blue arrows indicate a spermatocyte, spermatid, and sperm, respectively. Scale bar: 20 µm.

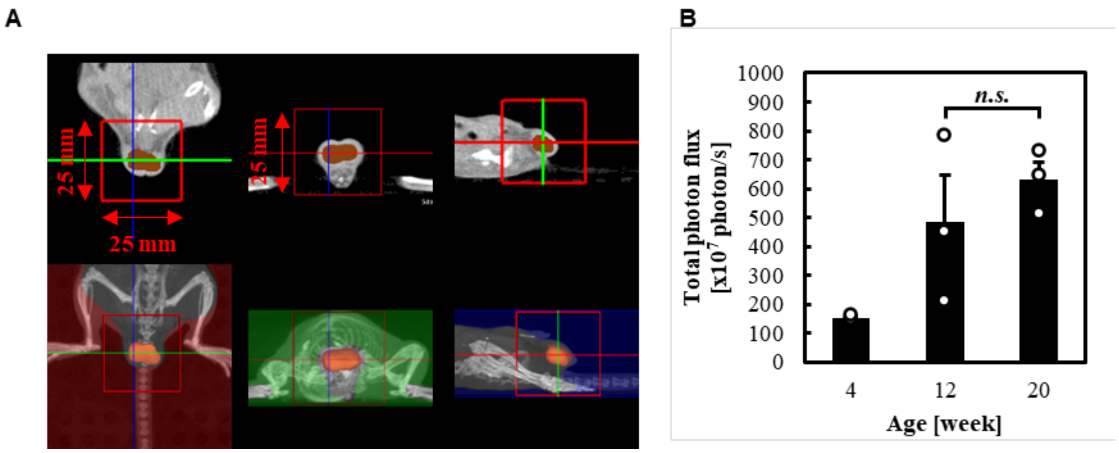

Figure S7. 3D bioluminescence imaging analysis of Acr-Luc KI mice.

(**A**) 3D reconstruction of testicular tissues generated from 2D bioluminescence images.

(**B**) Quantification of bioluminescence intensity in VOI at 4, 12, and 20 weeks of age. Data are presented as mean total photon flux ± SE from three mice; white dots indicate individual data points. *n.s.* = *not significant.*

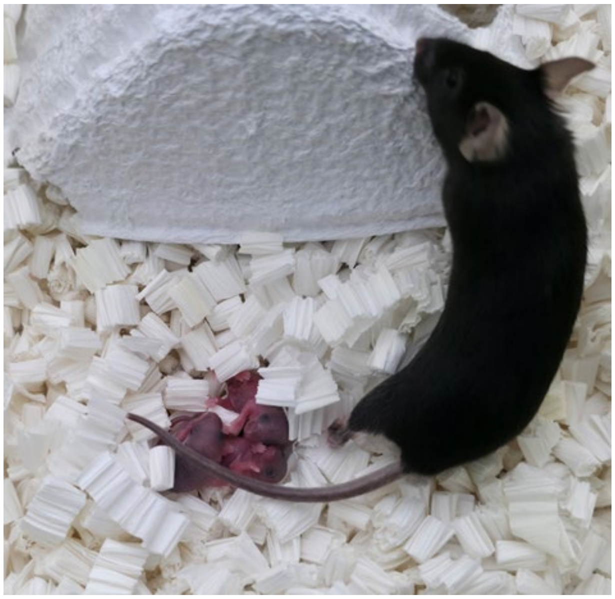

Figure S8. Pups of a 5 Gy-irradiated Acr-Luc knock-in mouse.

A 5 Gy-irradiated Acr-Luc knock-in mouse (corresponding to the “5 Gy-3” individual in Figure 4) was mated with a wild-type female 12 weeks after irradiation. The image shows the three pups born 24 days post-mating. No apparent morphological abnormalities or abnormal behaviors were observed in the pups.

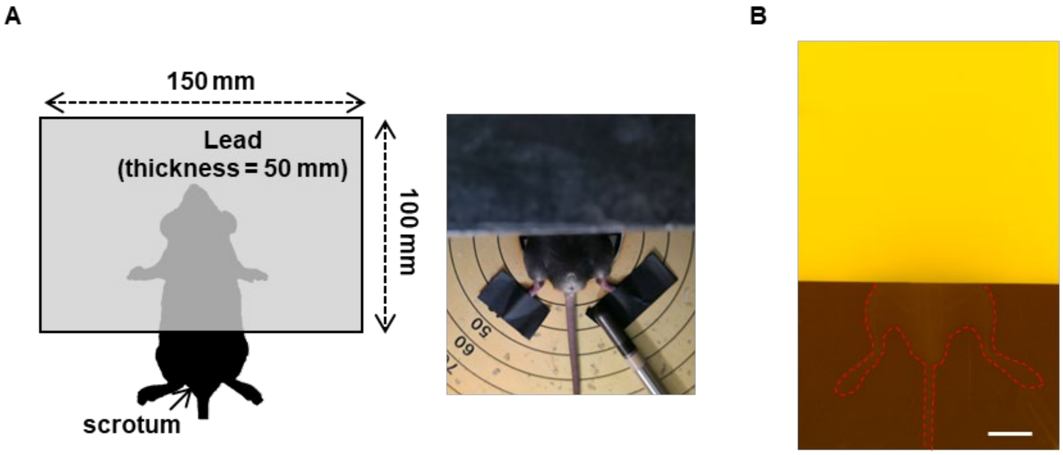

Figure S9. X-ray irradiation setup and dose verification for Acr-Luc KI mice.

(**A**) Schematic of the X-ray irradiation setup targeting the lower body of Acr-Luc KI mice. To ensure coverage of the testes despite physiological movement, the irradiation field included the region at and below the pelvic bones. Acr-Luc KI mice were anesthetized with isoflurane and exposed to 150 kVp X-rays. The exposure to the upper body was shielded using a lead (Pb) block ($15.0 \times 10.0 \times 5.0$ cm^3).

(**B**) Radiographic confirmation of the irradiation field using radiochromic film. The outline of the irradiated mouse body is indicated by a red dashed line. Scale bar: 10 mm.
